# Nematodes inhabiting the gastrointestinal tract and accessory organs of cricetid rodents in Argentina and Chile: a systematic review

**DOI:** 10.1017/S0031182026102029

**Published:** 2026-07

**Authors:** Marlon Mauricio Ardila, Iván Benavides-Céspedes, José D. Sarmiento-Roa, Richard Said Thomas, Adriana Santodomingo, Leidi Herrera, Pablo Oyarzún-Ruiz, AnaLía Henríquez, Sebastián Muñoz-Leal, Lucila Moreno, María del Rosario Robles, Carlos Landaeta-Aqueveque

**Affiliations:** 1 Departamento de Patología y Medicina Preventiva, Facultad de Ciencias Veterinarias, Universidad de Concepción, Chillán, Chile; 2 Departamento de Ciencia Animal, Facultad de Ciencias Veterinarias, Universidad de Concepción, Chillán, Chile; 3 Semillero de Investigación de Artrópodos Neoptera, Programa de Biología, Facultad de Ciencias Básicas, Universidad del Atlántico, Puerto Colombia, Colombia; 4 Grupo de Investigación de Producción y Salud en Medicina Veterinaria y Zootecnia (PROSAVEZ), Facultad de Ciencias Veterinarias y Zootecnia, Fundación Universitaria San Martín, Puerto Colombia, Colombia; 5 Centro de Investigación de Estudios Avanzados del Maule (CIEAM), Vicerrectoría de Investigación y Postgrado, Universidad Católica del Maule, Talca, Chile; 6 Instituto de Zoología y Ecología Tropical (IZET), Facultad de Ciencias, Universidad Central de Venezuela (UCV), Caracas, Venezuela; 7 Instituto de Investigaciones en Ciencias de la Salud, Universidad Nacional de Asunción, San Lorenzo, Paraguay; 8 Departamento de Microbiología, Facultad de Ciencias Biológicas, Universidad de Concepción, Concepción, Chile; 9 Facultad de Medicina Veterinaria, Universidad San Sebastián, Concepción, Chile; 10 Departamento de Zoología, Facultad de Ciencias Naturales y Oceanográficas, Universidad de Concepción, Concepción, Chile; 11 Centro Nacional de Investigación en Ríos, Invasiones y Sistemas (IRIS), Concepción, Chile; 12 Centro de Estudio Parasitológicos y de Vectores (CEPAVE), CCT-CONICET-La Plata/Universidad Nacional de La Plata, La Plata, Buenos Aires, Argentina

**Keywords:** biodiversity, Cricetidae family, helminths, nematode–rodent associations, neotropics, parasitic worms, small mammals

## Abstract

South American ecosystems harbour diverse cricetid rodents and their associated nematode parasites, with implications for ecology and zoonotic risk. We conducted a systematic review following the PRISMA guidelines to catalogue nematode *taxa* reported from the digestive tract and accessory organs of cricetid rodents in Argentina and Chile. Searches were carried out in five databases (PubMed, WoS, Scopus, SciELO, and Google Scholar) up to 10 January 2026. Parasitological descriptors, such as prevalence (P), mean abundance (_M_A), and mean intensity (_M_I) were estimated. We analysed 53 studies examining 4400 rodents (33 species), with *Abrothrix olivacea* (*n* = 1000 specimens) and *Akodon azarae* (*n* = 775 specimens) most frequently sampled. Ninety-two nematode *taxa* were reported, with *A. olivacea* exhibiting the highest richness (17 *taxa*). *Guerrerostrongylus zeta* showed the highest P (97.56% in *Oligoryzomys nigripes*), followed by *Stilestrongylus stilesi* (92.5% in *Necromys lasiurus*) Peak infection intensities occurred with *Stilestrongylus flavescens* in *O. flavescens* (_M_A = 72.79/_M_I = 126.7) and *St. stilesi* in *N. lasiurus* (_M_A = 120/_M_I = 129.73). This review provides the first comprehensive synthesis of gastrointestinal and hepatopancreatic nematodes in cricetid rodents from Argentina and Chile, highlighting research biases and priorities for advancing our understanding of nematode–cricetid rodent dynamics in the region. The overrepresentation of *A. olivacea* underscores the need to expand the range of cricetid rodent sampled and to increase the use of non-invasive and molecular diagnostic tools. Addressing these gaps will improve our understanding of nematode diversity and associated zoonotic risks in cricetid rodents in these countries.

## Introduction

Rodents (Mammalia: Rodentia) are the most diverse, abundant, and geographically widespread order of mammals, representing nearly 40% of all mammalian species (Patton et al., 2015; Wilson et al., 2017; D’Elía et al., 2019; Burgin et al., 2025; Hernández-Mazariegos et al., 2025; Mammal Diversity Database, 2026). Many rodent species possess traits that can facilitate the maintenance and transmission of a variety pathogens (e.g., viruses, bacteria, unicellular eukaryotic parasites, and parasitic helminths), which has led to their recognition as important reservoir hosts capable of driving spillover events to other wildlife, domestic animals, or humans (Han et al., 2015; Lacher et al., 2017; Landaeta-Aqueveque et al., 2021).

Such traits, which are not uniformly present across all rodent *taxa*, may include the ability to occupy a wide range of ecological niches, fast life histories characterized by early sexual maturation and high reproductive output, high population densities and rapid population turnover, diverse activity patterns, broad and flexible diets, marked behavioural plasticity, high dispersal capacity and the facility of many species to thrive in human-modified environments (synanthropy) (Singla et al., 2008; Karesh et al., 2012; Han et al., 2015; Wilson et al., 2017). Among rodents, those belonging to the family Cricetidae comprise one of the most diverse and widely distributed groups in the Americas, with approximately 70 genera and 451 species in South America (hereafter, SA) alone (Patton et al., 2015; D’Elía et al., 2019; Teta et al., 2025).

In the southern cone of SA, Argentina and Chile together cover a vast territory that includes a wide variety of biogeographic regions, ranging from Patagonian steppes and temperate rainforests to Mediterranean scrublands and high Andean environments, many of which are recognized as biodiversity hotspots (Morrone, 2014, 2015; Patton et al., 2015). This diversity is closely linked to the broad latitudinal and climate gradients that characterized both countries. In Chile, climate ranges from arid in the north (e.g., Atacama Desert) through Mediterranean in the central regions to temperate and subpolar (in the south). Similarly, Argentina spans subtropical zones in the northeast, semi-arid zones in the northwest and Patagonia, temperate zones in the Pampas, and subpolar zones in the southern Patagonia and Tierra del Fuego (Di Castri, 1968; Di Castri and Hajek, 1976; Garreaud et al., 2009; Morello et al., 2012; Robles and Navone, 2014; Ministerio del Medio Ambiente, 2018; Fugassa, 2020).

The environmental mosaic present in both countries supports high diversity and endemism of cricetid rodents, with 107 species documented in Argentina, 27 of which are endemic and 42 species recorded in Chile, including nine endemic species (D’Elía et al., 2020; Iriarte, 2021; Teta et al., 2021, 2025; Hernández-Mazariegos et al., 2025). These small mammals play an important ecological role in the ecosystems of both countries by modifying soil structure through burrowing and influencing plant community composition via seed consumption and dispersal (Han et al., 2015; Lacher et al., 2017; Hernández-Mazariegos et al., 2025). In addition, they constitute key prey for native predators (e.g., foxes, owls, hawks, and falcons) and can carry parasites that affect animal and human health (Lacher et al., 2017; Hernández-Mazariegos et al., 2025).

In rodents, nematodes are one of the most frequently reported helminths parasite groups (Landaeta-Aqueveque et al., 2021; Miljević et al., 2022). The ecological and life-history traits of these small mammals facilitate exposure and transmission of diverse nematodes across different hosts and habitats (Pagel et al., 1991; Singla et al., 2008; Karesh et al., 2012; Han et al., 2015; Wilson et al., 2017; Preisser and Falcón-Ordaz, 2019; Gómez-Muñoz et al., 2020). Nematodes commonly inhabit the gastrointestinal tract, where they may influence host nutrition, health, and population dynamics involving effects on fitness and survival (Hudson et al., 1998; Marcogliese, 2004; Landaeta-Aqueveque et al., 2007; Poulin 2007; Lafferty et al., 2008; Panisse et al., 2017; Cardoso et al., 2018; Costa et al., 2022; Carrera-Játiva et al., 2023).

Beyond the gastrointestinal tract, however, several nematodes are also capable of infecting accessory organs associated with the digestive system, like the liver, causing tissue damage and possible altering host physiology (Fantozzi et al., 2018, 2019). The pancreas may represent another potential target for nematodes given its close anatomical and functional connections with the gastrointestinal tract, including shared ductal systems and vascular networks (Shaik et al., 2025), which may render it physiologically susceptible to parasitic invasion under specific conditions, such as high parasite loads, atypical parasite migration or systemic larval dissemination (Bain and Junker, 2013; Barbosa-Valenzuela et al., 2021; Regmi et al., 2024; Chang et al., 2025).

In Argentina and Chile, nematodes are the best-documented helminth component in cricetids, from both taxonomic and ecological perspectives (Fugassa, 2020; Landaeta-Aqueveque et al., 2021). Several studies have shown that theses parasites influence host health, population dynamics, and ecological interactions (Miño, 2008; Fantozzi et al., 2018, 2019; Landaeta-Aqueveque et al., 2021; Palavecino et al., 2023; Hernández-Mazariegos et al., 2025). Moreover, some cricetid species occur syntopically in these countries (i.e., sharing the same microenvironment at the same time) (Courtalon and Busch, 2010; Cirignoli et al., 2011; García et al., 2013; Udrizar-Sauthier and Pardiñas, 2014; Astorga et al., 2018; Landaeta-Aqueveque et al., 2018; Salgado et al., 2022; Vadell et al., 2024; Quiroga-Carmona et al., 2025), potentially facilitating nematode transmission among different host species (Landaeta-Aqueveque et al., 2018).

Given this syntopic occurrence, along with the marked environmental heterogeneity and the high diversity of cricetid rodents in both countries, nematode assemblages likely vary among host species and geographic areas. However, despite this ecological context, the available information has not yet been integrated into a comprehensive regional synthesis. Consequently, current knowledge on nematode diversity across digestive tract and accessory rodents from Argentina and Chile remains fragmented.

Although some nematode checklists are available for rodents in SA (e.g., Fugassa, 2020; Landaeta-Aqueveque et al., 2021), these compilations often do not capture the full diversity of cricetid-associated nematodes or incorporated the latest taxonomic and methodological advances in the field (Pinto et al., 2011; Robles and Navone, 2014; Fugassa, 2020; Landaeta-Aqueveque et al., 2021), particularly for Argentina and Chile. In this sense, a comprehensive review of these parasites inhabiting the gastrointestinal tract and accessory organs of cricetid rodents in Argentina and Chile is, therefore, necessary to consolidate current knowledge, identify research gaps, and provide a baseline for future ecological, parasitological, and epidemiological studies in the southern of SA.

Accordingly, we conducted a systematic review of the scientific literature on nematodes associated with the digestive tract and accessory organs of cricetid rodents in Argentina and Chile to achieve the following objectives: (i) analyse the occurrence and geographical distribution of nematodes species reported in cricetid rodents; (ii) explore patterns of infection using parasitological descriptors, including prevalence (P), mean abundance (_M_A), and mean intensity (_M_I); and (iii) describe and synthesize the methodological approaches used to study nematode infections in cricetid rodents.

Quantifying parasite infection parameters, such as P, _M_A and _M_I, is pivotal to understanding nematode–host association patterns and infection dynamics (Bush et al., 1997). These descriptors enable comparisons among host species and geographic regions and provide insights into parasite transmission, host susceptibility, and the influence of environmental factors on parasite communities (Rózsa et al., 2000; Poulin, 2007). Collectively, the analysis of these parameters helps clarify ecological patterns of nematode–cricetid associations across different environments in the southern SA (Marcogliese, 2004; Landaeta-Aqueveque et al., 2018).

By integrating taxonomic, ecological and methodological information, this review aims to provide a comprehensive framework for understanding nematode–cricetid associations in southern SA and to highlight priorities for future research in the region.

## Materials and methods

### Study design and scope

A systematic review was conducted to evaluate and synthesize the records of nematode fauna associated with cricetid rodents in Argentina and Chile. Other helminth groups (e.g., cestodes, trematodes) are not considered here and fall outside the scope of this study. The review adhered to the conceptual and methodological aspects of the PRISMA (Preferred Reporting Items for Systematic Reviews and Meta-Analyses) statement to maximize methodological transparency and minimize potential biases in the results (Page et al., 2021).

This review targeted studies reporting natural infection by parasitic nematodes in cricetid rodents, with infections occurring in the digestive tract (from oesophagus to the rectum) and related accessory organs (e.g., the liver and pancreas) in the study area. Studies on nematodes that inhabit the thoracic or pleural cavities (e.g., filarial nematodes) were explicitly excluded, because they are outside the scope of this review, which focuses on the gastrointestinal tract and associated organs.

### Search strategies

The literature search was conducted using the PubMed (https://pubmed.ncbi.nlm.nih.gov/), Web of Science (https://www.webofscience.com/wos/woscc/basic-search), Scopus (https://www.scopus.com/search/form.uri?display=basic#basic), SciELO (Scientific Electronic Library Online, https://scielo.org/es/), and Google Scholar (https://scholar.google.com/) databases. The Boolean operator ‘AND’ was used to refine the search strategy, along with the following search terms: ‘Cricetidae AND gastrointestinal helminths’, ‘Rodent AND gastrointestinal helminths’, ‘Rodent AND Endoparasites’, ‘Cricetidae AND endoparasites’, ‘Rodentia AND Nematoda AND Argentina’ and ‘Rodentia AND Nematoda AND Chile’. In addition, organ-specific terms such as ‘liver’, ‘hepatic’, and ‘pancreas’ were included due to the consideration of accessory organs in the review. Searches were conducted in both English and Spanish languages and were limited to scientific articles published up to 10 January 2026.

#### Study selection criteria

Study selection was conducted in two phases. First, titles and abstracts were screened to identify papers that explicitly included the search terms. Later, duplicated records were identified and removed, and only one version of each article was retained. Abstracts were further reviewed to ensure the relevance of the study topic. Second, full texts were reviewed for eligibility based on the following criteria: publication in peer-reviewed journals up to 10 January 2026, written in English or Spanish, and focused on parasitic nematodes infecting cricetid rodents in Argentina and Chile.

A backward citation search (snowballing) was performed by reviewing the reference lists of all selected documents and relevant review papers to identify primary studies not retrieved through the database search, and studies meeting the predefined selection criteria were added to the final dataset. Grey literature (e.g., theses and reports), books, book chapters, conference proceedings, poster abstracts, experimental studies, and review articles were excluded. Review articles were used solely to identify eligible primary studies. Only observational studies reporting natural nematode infections in cricetid rodents were considered.

### Data extraction and processing

A dataset was compiled using Microsoft Excel^®^ 2019, incorporating all studies retrieved from the selected databases. From each eligible study, an internal identification code was assigned (for internal referencing) and the following information was systematically extracted from the papers: year of publication and authors, country and specific study location (province or region), rodent species examined for nematodes, nematode species identified and identification methods, site of nematode infection, sample size of rodents analysed, and the number of positive cases per nematode taxon. Parallelly, to ensure up-to-date and accurate taxonomic identification of rodent hosts, species names and classifications were cross-checked and updated according to the Mammal Diversity Database: https://www.mammaldiversity.org/search/.

### Mapping the geographical distribution and occurrence of nematode-associated cricetid rodents

To analyse the geographical distribution of nematodes reported in cricetid rodents, location data were extracted from the selected studies. When precise geographical coordinates were unavailable, approximate locations were inferred based on the description of the study areas (e.g., province or region).

Geographical information was organized in a structured database to ensure consistency in the locality nomenclature. Spatial analyses and map generation were performed using QGIS v3.44.0-Solothurn. Base layers consisted of administrative boundaries of Argentina and Chile, obtained from the open-access sources Natural Earth datasets (https://www.naturalearthdata.com/). The distribution of nematodes and their cricetid hosts was represented using point markers, with each point corresponding to at least one record of a nematode species in cricetid rodents at a given locality.

At each sampling site, a pie chart was overlaid to illustrate the relative composition of cricetid species present. A unified colour-coding scheme linked to a detailed legend was applied across all maps. The size of each pie chart was scaled according to the total number of cricetid species recorded at each site, allowing visual comparison of local richness and community structure. For analytical purposes, ‘diversity’ was operationalized as component-community nematode taxon richness, defined as the number of distinct nematode *taxa* recorded across the set of hosts sampled per locality (Bush et al., 1997). When nematodes were identified above the species level, richness estimates were based on the finest taxonomic resolution reported in each study.

This mapping approach facilitates the visualization of spatial patterns in nematode occurrence in relation to host richness and distribution. The resulting maps provide a regional overview of nematode diversity and distribution across the study area, enabling comparative interpretation of geographical patterns at a regional scale.

### Estimation of parasite infection rates

An Excel-based matrix was used to compile the total number of hosts examined, the number of infected hosts and the number of nematodes recorded in each of the studies. Based on these data, the following parasitological descriptors were calculated for each nematode taxon: P%: number of parasitized hosts/number of hosts examined, × 100; _M_A: number of individuals of a parasitic species/number of hosts examined; and _M_I: number of individuals of a parasitic species/number of infected hosts. To minimize potential bias, only studies that analysed more than 30 rodents were included in the estimation of these descriptors (Bush et al., 1997).

To further characterize host–parasite associations, analyses focused on the three nematode genera with the highest species richness identified in the dataset. For each genus, the frequency of occurrence of constituent species across all documented rodent hosts was recorded. In addition, the number of cricetid species associated with each nematode taxon was quantified. This approach allowed the assessment of host range and specificity patterns among the most diverse nematode genera infecting cricetid rodents in Argentina and Chile.

## Results

### General findings

**Figure 1.**
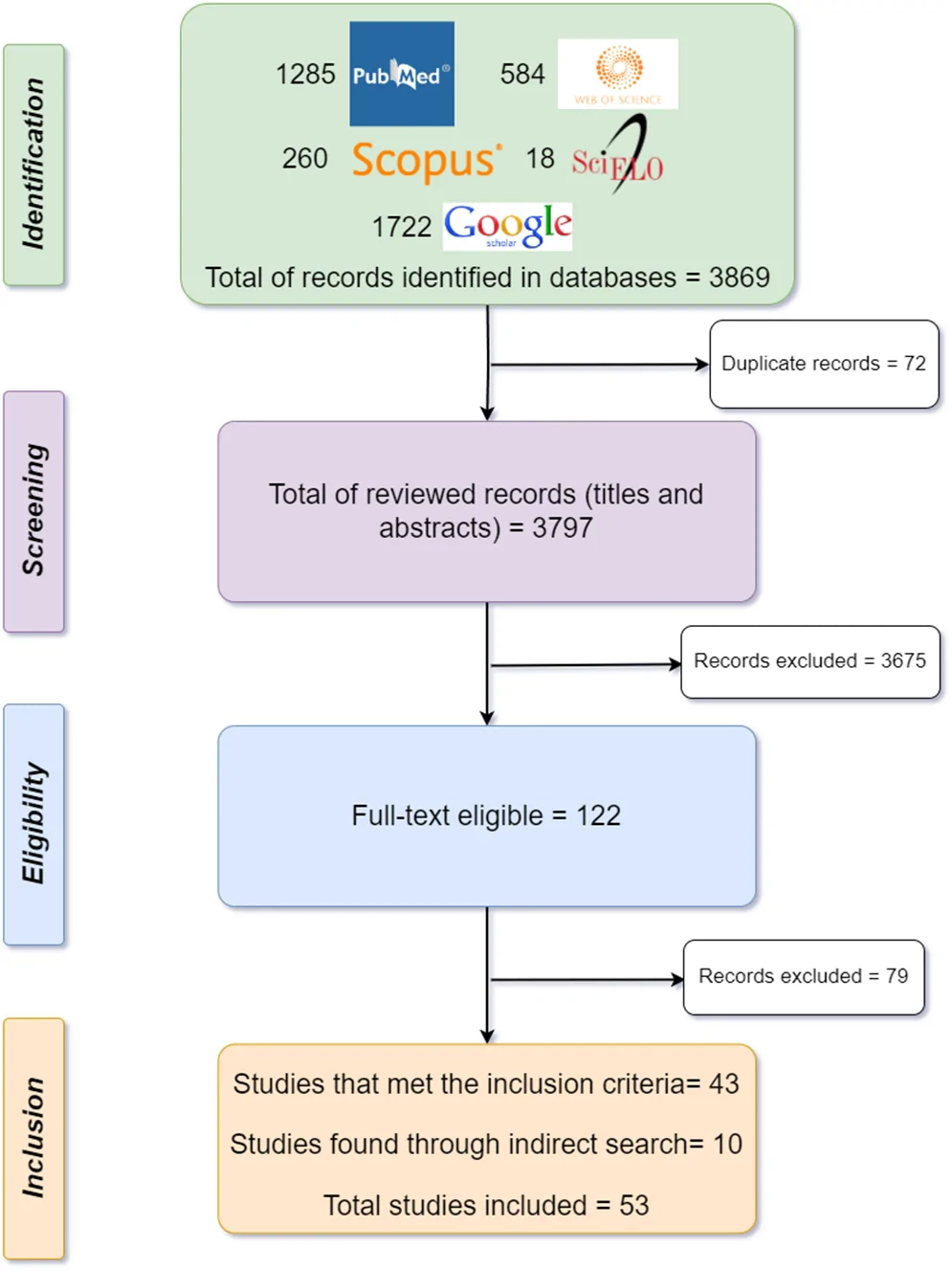
Flow diagram showing the selection procedure for study inclusion in the systematic review (PRISMA method). Figure 1 long description.

The search across the databases yielded 3869 studies: 1285 from PubMed, 584 from WoS, 260 from Scopus, 18 from SciELO, and 1722 from Google Scholar. After removing 72 duplicates, 3797 unique records remained (Figure 1). Titles and abstracts screening excluded 3675 articles, leaving 122 studies for full-text review. Of these, 79 studies failed to meet the inclusion criteria and were excluded, resulting in 43 eligible studies (Figure 1). Backward citation tracking of included studies and relevant reviews identified 10 additional studies, bringing the final dataset to 53 studies (Figure 1).

### Rodent species examined for nematodes

**Figure 2.**
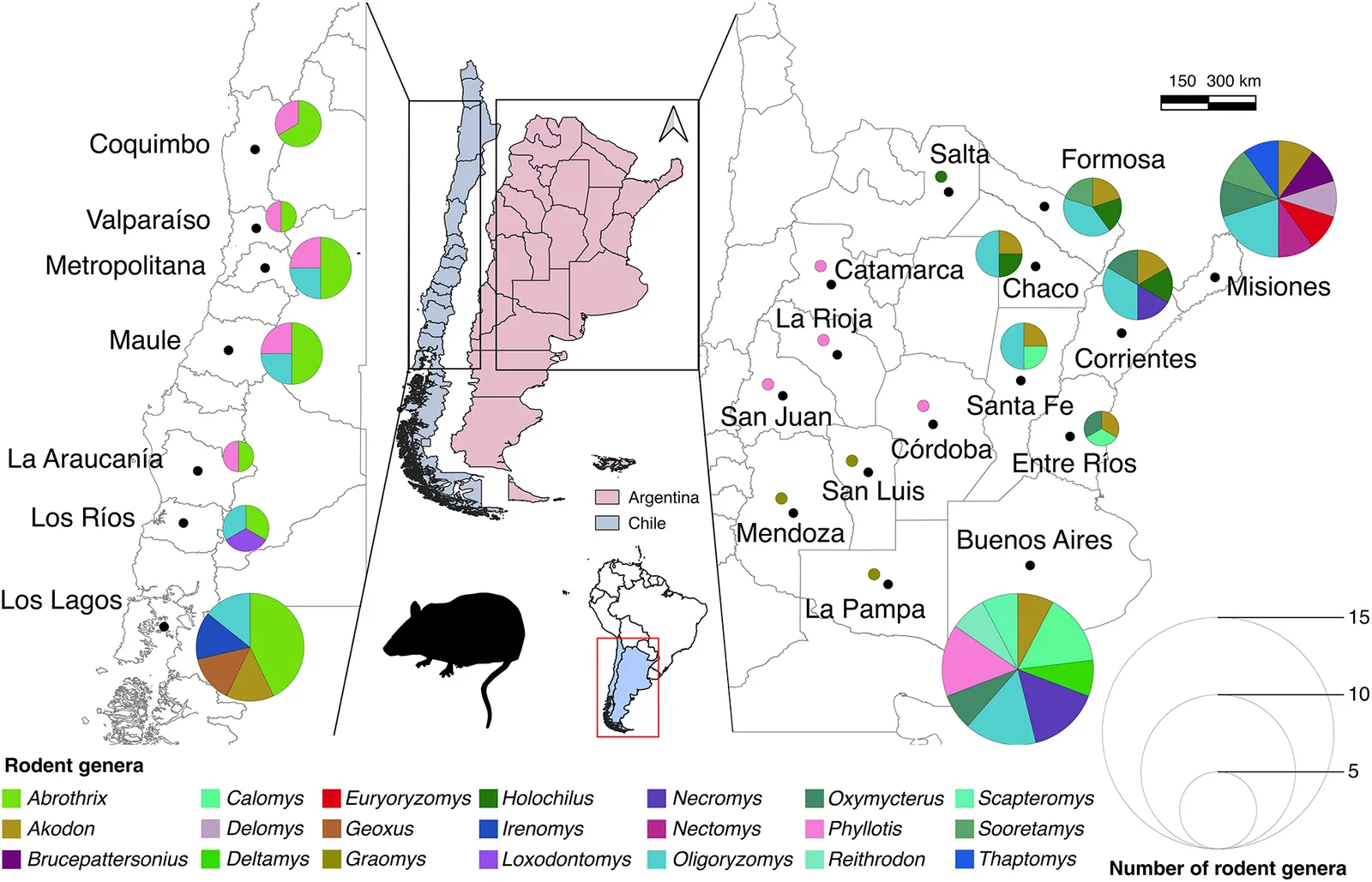
Geographical distribution and local composition of cricetid rodent genus richness in Argentina and Chile. Map of Argentina and Chile highlighting surveyed localities, reporting nematode records in cricetid rodents detected at each site. Each pie chart represents the relative proportion of rodent genera recorded at each locality, according to the colour key linked to the genera listed on the right. The size of each pie chart is proportional to the total number of rodent genera at that locality. The reference in the lower right corner indicates the correspondence between pie chart size and the number of genera. The map inset shows the study area within the South American context. Figure 2 long description.

A total of 21 rodent genera were examined for nematodes across the 53 selected studies, with records spanning most Argentinian provinces and several Chilean regions (Figure 2, Supplementary Table S1). The highest rodent genus richness was observed in the Buenos Aires and Misiones (nine genera each) provinces in Argentina and in the Los Lagos region in Chile (five genera each). Overall, 4400 rodents were evaluated, though six studies (11.32%) did not report the exact number of rodents collected (Supplementary Table S1).

Among the analysed rodents, 98.93% (4353 individuals) were identified to species rank, representing 33 host species (Supplementary Table S1). The most frequently sampled rodents were *Abrothrix olivacea* (*n* = 1000), followed by *Akodon azarae* (*n* = 775), *Phyllotis darwini* (*n =* 527), *Scapteromys aquaticus* (*n* = 362), and *Abrothrix longipilis* (*n* = 317) (Supplementary Table S1). In contrast, the remaining 47 individuals (1.06%) were identified only to the genus rank: *Akodon* sp. with 23 individuals, *Phyllotis* sp. with 17 individuals and *Brucepattersonius* sp. with seven individuals (Supplementary Table S1).

### Nematode species recorded in cricetid rodents

Ninety-two nematode *taxa* were reported across the analysed studies (Supplementary Table S2). Of these, 64 *taxa* (69.57%) were identified to the species rank, 17 *taxa* (18.48%) to the genus rank, one taxon (1.08%) to the subfamily rank, four *taxa* (4.35%) to the family rank, two *taxa* (2.17%) to superfamily rank, two *taxa* (2.17%) to the suborder rank and two *taxa* (2.17%) to the order rank (Supplementary Table S2). Regarding anatomical location, 44 *taxa* were recovered from the small intestine, 25 from the large intestine (e.g., cecum, colon, and rectum), 15 from the stomach, and one from the liver (Supplementary Table S2). Conversely, some studies analysed nematodes from 1647 rodent faecal samples (Supplementary Table S2). No records of nematodes associated with the pancreas of cricetid rodents were found in the reviewed papers.

The rodent *A. olivacea* had the highest richness of nematode *taxa* (*n =* 17), followed by *P. darwini* (*n* = 14), and *S. aquaticus* (*n* = 13). In contrast, *Abrothrix sanborni, Calomys callidus, Calomys laucha, Calomys venustus, Delomys dorsalis, Loxodontomys micropus, Nectomys obscurus, Oxymycterus quaestor, Phyllotis bonariensis,* and *Reithrodon auritus,* each harboured only a single nematode species (Supplementary Table S2).

In terms of parasite host range, the nematode species *Calodium hepaticum* was found in the highest number of host species (six rodent species). *Stilestrongylus azarai, Syphacia carlitosi,* and *Trichuris laevitestis* were the most abundant nematodes, with 152, 156, and 123 individuals recorded, respectively (Supplementary Table S3).

### Geographical patterns of nematode–cricetid rodent associations

**Figure 3.**
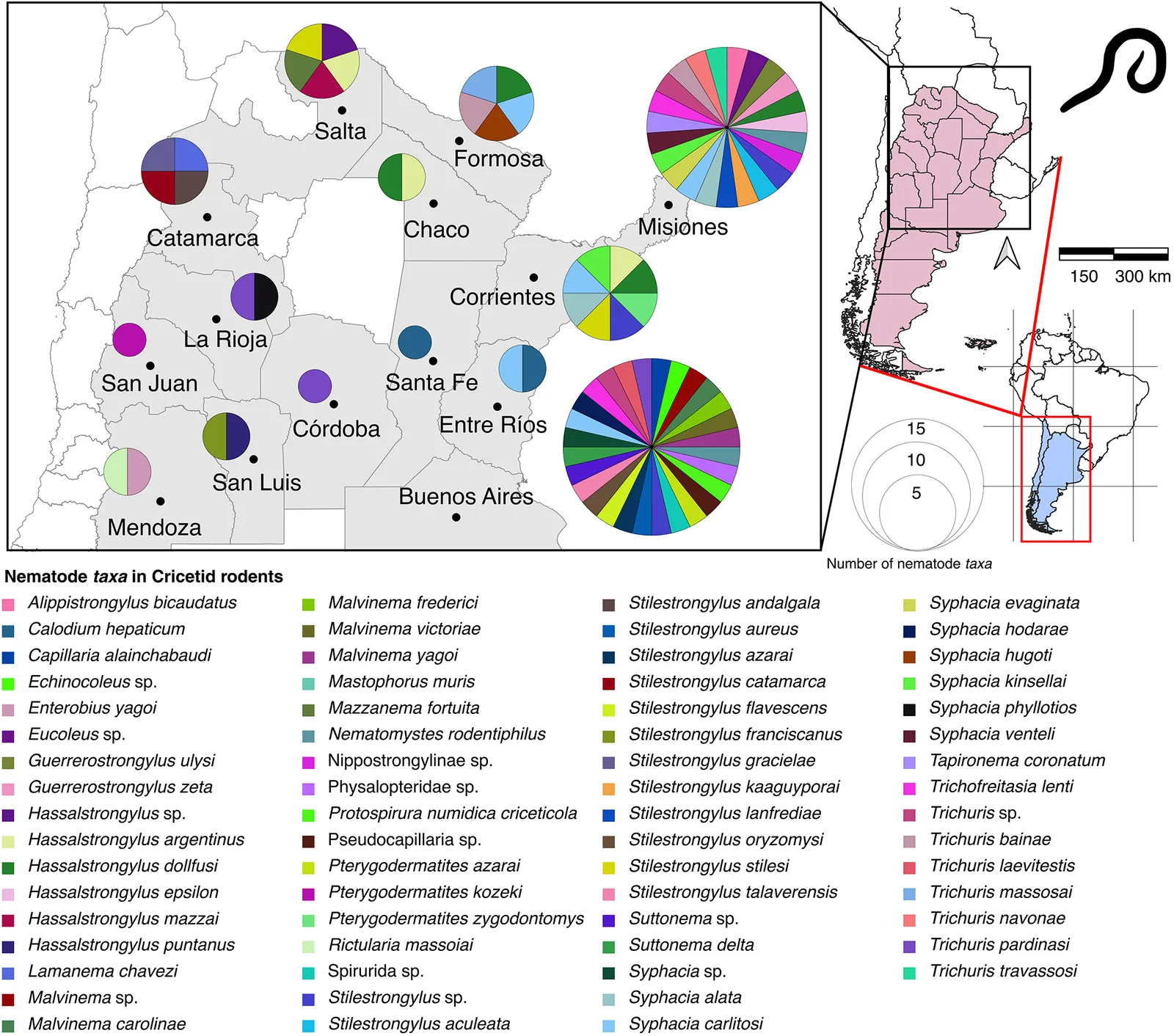
Geographical distribution and local composition of nematode *taxa* richness in cricetid rodents in Argentina. Map of Argentina highlighting surveyed localities, showing the richness and composition of nematode *taxa* species detected in populations of cricetid rodents. Each pie chart represents the relative proportion of nematode *taxa* recorded at each locality, according to the colour key linked to the *taxa* listed below. The size of each pie chart is proportional to the total number of nematode *taxa* detected at that site. The reference in the lower left corner indicates the correspondence between the size of the pie charts and the number of *taxa* found. The list below presents the nematode *taxa* recorded and their corresponding colours. Figure 3 long description.

**Figure 4.**
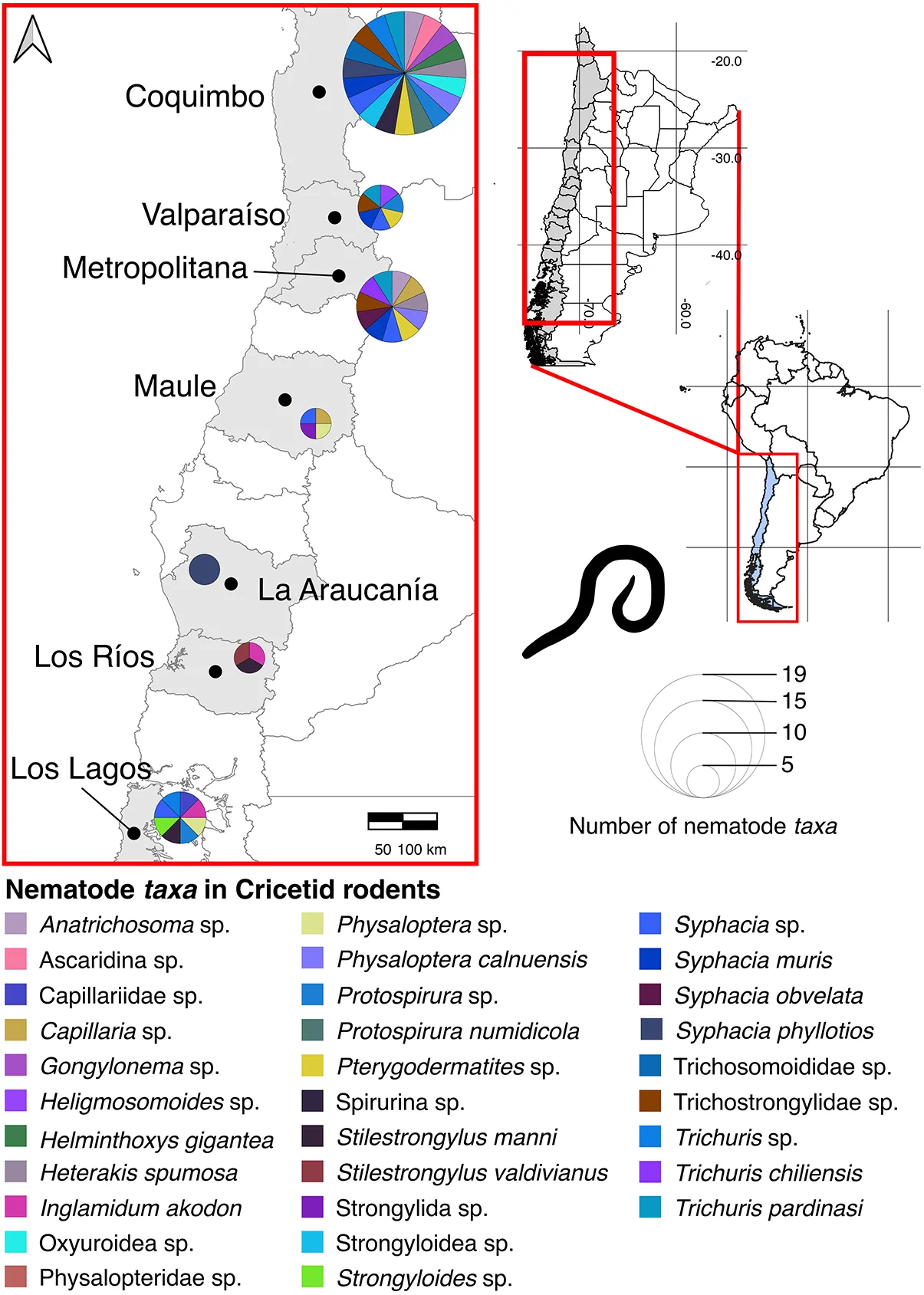
Geographical distribution and local composition of nematode *taxa* richness in cricetid rodents in Chile. Map of Chile highlighting surveyed localities, showing the richness and composition of nematode *taxa* species detected in populations of cricetid rodents. Each pie chart represents the relative proportion of nematode species recorded at each locality, according to the colour key linked to the taxa listed below. The size of each pie chart is proportional to the total number of nematode species detected at that site. The reference in the lower left corner indicates the correspondence between the size of the pie charts and the number of species found. The list below presents the nematode taxa recorded and their corresponding colours. Figure 4 long description.

Nematodes richness associated with cricetid rodents varied markedly across the surveyed localities (Figures 2–4). In Argentina, Buenos Aires (*n* = 28 parasitic *taxa*) and Misiones (*n* = 23 parasitic *taxa*) provinces harboured the highest nematode taxon richness, whereas La Pampa, San Juan, Córdoba and Santa Fe provinces exhibited poorer assemblages (one parasitic *taxa* each) (Figure 3). In Chile, the Coquimbo (*n* = 19 parasitic *taxa*) and Metropolitana (*n* = 11 parasitic *taxa*) regions harboured the highest nematode taxon richness, whereas the La Araucanía region exhibited lower assemblages (*n* = 1 parasitic *taxa*) (Figure 4).

**Figure 5.**
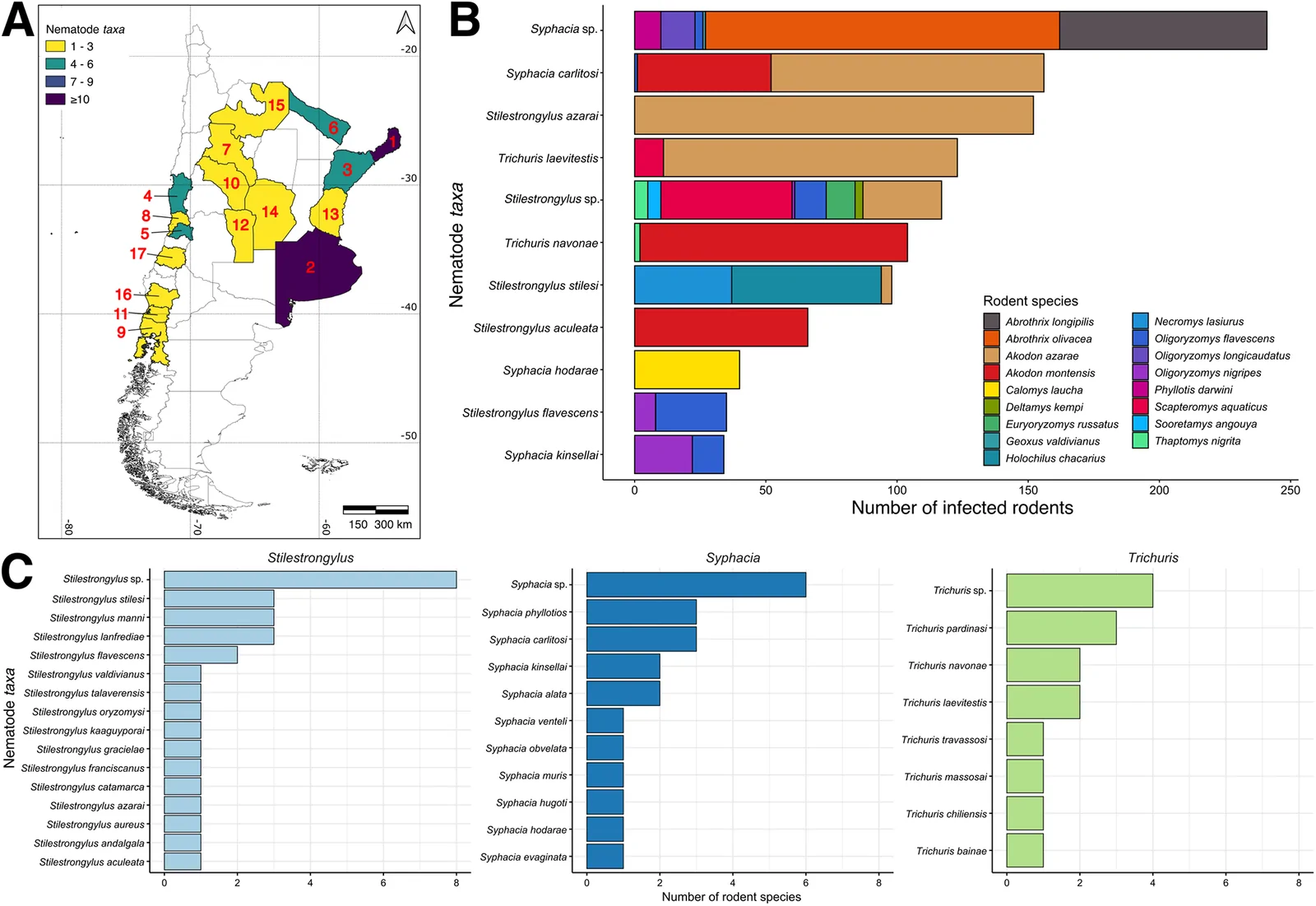
Diversity, distribution and host–parasite associations of nematodes of the genera *Stilestrongylus, Syphacia* and *Trichuris* in cricetid rodents in Argentina and Chile. (A) Map showing the species richness of these nematodes by province or region in Argentina and Chile, respectively, according to the number of species recorded (see colour scale). Red numbers indicate the surveyed localities included in the study. Surveyed localities: 1. Misiones province, 2. Buenos Aires province, 3. Corrientes province, 4. Coquimbo region, 5. Metropolitana region, 6. Formosa province, 7. Catamarca province, 8. Valparaíso region, 9. Los Lagos region, 10. La Rioja province, 11. Los Ríos region, 12. San Luis province, 13. Entre Ríos province, 14. Córdoba province, 15. Salta province, 16. La Araucanía region, 17. Maule region. (B) Frequency of occurrence of the main nematode *taxa* across different rodent hosts, with each bar coloured according to the associated rodent species (see legend at right). (C) Number of rodent host species associated with each nematode *taxa*, allowing for the description of host range and specificity. Figure 5 long description.

Nematode taxon richness per localities ranged from one to 13 parasitic *taxa*, though most sites (64.71%; *n* = 11) harboured only one to three *taxa,* whereas two sites showed more than 10 parasitic *taxa* (Figure 5A). Within the assemblage of nematodes, *Sy. carlitosi, St. azarai,* and *T. laevitestis* were the most frequently encountered species in the examined material (Figure 5B). Among these, *Sy. carlitosi* and *T. laevitestis* infected the highest number of rodent species, parasitizing three rodent species (*A. azarae, Akodon montensis,* and *Oligoryzomys flavescens*) and two rodent species (*A. azarae* and *S. aquaticus*), respectively, whereas *St. azarai* was recorded primarily in a single host species (*A. azarae*) (Figure 5B–C).

### Methods of investigating and techniques used for the study of nematodes in cricetid rodents

The majority of studies (92.5%; *n* = 49) identified nematodes through necropsies, including both opportunistic and targeted dissections. A smaller proportion (5.7%; *n* = 3) used coprological technique involving optical microscopy with the modified Telemann technique, the modified Ritchie technique, and the Mini-FLOTAC technique. Only one study included coprological analysis and necropsy for examined different individuals (1.9%) (Table 1).

**Table 1.** Nematodes of the gastrointestinal tract and accessory organs in cricetid rodents from Argentina and Chile: summary of parasitological studies by sample type and diagnostic technique Table 1 long description.

References	Sample type	Diagnostic technique
Digiani and Kinsella (2014)	Necropsy	OM/SEM
Digiani et al. (2003)	Necropsy	OM
Robles et al. (2006)	Necropsy	OM/SEM
Panisse and Digiani (2018)	Necropsy	OM
Robles and Navone (2007a)	Necropsy	OM/SEM
Rojas-Herrera et al. (2011)	Necropsy	OM/SEM
Robles and Navone (2007b)	Necropsy	OM/SEM
Robles et al. (2014a)	Necropsy	OM/SEM
Fantozzi et al. (2018)	Necropsy	OM/H/Mo^a^
Babero et al. (1976)	Necropsy	OM
Robles et al. (2012)	Necropsy	OM/SEM
Miño et al. (2020)	Coprological	OM^b^
Navone et al. (2009)	Necropsy	OM
Cattan et al. (1992)	Necropsy	OM
Durette-Desset and Sutton (1985)	Necropsy	OM
Sutton (1979)	Necropsy	OM
Sutton et al. (1980)	Necropsy	OM
Sutton (1989)	Necropsy	OM
Landaeta-Aqueveque et al. (2007)	Necropsy	OM
Digiani and Durette-Desset (2003a)	Necropsy	OM
Panisse et al. (2017)	Necropsy	OM
Miño et al. (2012)	Necropsy	OM
Riquelme et al. (2021)	Coprological	OM^c^
Robles (2011)	Necropsy	OM/SEM
Suriano and Navone (1994)	Necropsy	OM
Carrera-Játiva et al. (2023)	Necropsy/coprological	OM^d^
Miño (2008)	Necropsy	OM
Digiani and Durette-Desset (2007)	Necropsy	OM
Miño et al. (2019)	Necropsy	OM
Yáñez-Meza et al. (2019)	Necropsy	OM
Digiani and Durette-Desset (2003c)	Necropsy	OM
Digiani et al. (2015)	Necropsy	OM
Digiani et al. (2013)	Necropsy	OM
Castillo et al. (2016)	Necropsy	OM
Robles and Navone (2010)	Necropsy	OM/SEM
Landaeta-Aqueveque et al. (2018)	Necropsy	OM
Hancke and Suarez (2018)	Necropsy	OM
Robles and Navone (2006)	Necropsy	OM/SEM
Notarnicola et al. (2010)	Necropsy	OM
Rojas and Digiani (2003)	Necropsy	OM/SEM
Quentin et al. (1979)	Necropsy	OM
Durette-Desset et al. (1976)	Necropsy	OM
Sutton and Durette-Desset (1991)	Necropsy	OM
Durette-Desset and Murua (1979)	Necropsy	OM
Denke and Murua (1977)	Necropsy	OM
Digiani et al. (2012)	Necropsy	OM
Gómez-Muñoz et al. (2020)	Necropsy	OM
Chemisquy et al. (2021)	Necropsy	OM
Digiani and Durette-Desset (2003b)	Necropsy	OM
Robles et al. (2014b)	Necropsy	OM/SEM/Mo^e^
Robles et al. (2018)	Necropsy	OM/SEM/Mo^f^
Carrera-Játiva et al. (2025)	Coprological	OM^g^
Serrano et al. (2021)	Necropsy	OM

OM, optical microscopy; SEM, scanning electron microscopy; H, histology; Mo, molecular approach.

a *18S rRNA* sequencing.

b Coprological with the modified Ritchie technique.

c Coprological with the modified Telemann.

d Coprological with the Mini-FLOTAC.

e ITS2 sequencing.

f *cox1, cob,* and ITS2 sequencing.

g Coprological with the Mini-FLOTAC.

Among necropsy-based studies (*n* = 50), 26% (*n* = 13) employed scanning electron microscopy (SEM) for detailed morphological characterization. In contrast, integrative approaches were rare: one study (2%) combined histology with sequencing of the small subunit ribosomal RNA (*18S rRNA)* marker; another (2%) used the mitochondrial markers cytochrome c oxidase subunit I (*cox1*) and cytochrome b (*cob/cytb*), along with the nuclear internal transcribed spacer 2 (ITS2) marker and 1 (2%) employed only ITS2. The remaining 68 (*n* = 34) relied exclusively on light microscopy for taxonomy identification (Table 1).

### Nematode infection rates in cricetid rodents

High P values were observed for some nematode species in their historical hosts. For example, *Guerrerostrongylus zeta* showed a P of 97.56% (40/41) in *Oligoryzomys nigripes*, followed by *Stilestrongylus stilesi* with 92.5% (37/40) in *Necromys lasiurus,* and *Hassalstrongylus dollfusi* with 91.11% (41/45) in *O. flavescens.* The _M_A and _M_I varied across rodent and nematode species. The highest values were recorded for *Stilestrongylus flavescens* in *O. flavescens* (_M_A = 72.79 and _M_I = 126.7) and *St. stilesi* in *N. lasiurus* (_M_A = 120 and _M_I = 129.73) (Table 2).

**Table 2.** Nematodes recorded in the gastrointestinal tract and accessory organs from cricetid rodents in Argentina and Chile Table 2 long description.

Nematode *taxa*	Rodent (species)	Rodent sample size	Number of positive rodents for nematodes	P (%)	MA	MI	References
*Anatrichosoma* sp.	*Abrothrix olivacea*	119	4	3.36	0.73	21.75	Landaeta-Aqueveque et al., 2018
Ascaridina sp.	*Phyllotis darwini*	363	11	3.03	N/A	N/A	Carrera-Játiva et al., 2025
Capillariidae sp.	*Abrothrix olivacea*	134	8	5.97	N/A	N/A	Carrera-Játiva et al., 2023
*Calodium hepaticum*	*Akodon azarae*	145	51	35.17	N/S	N/S	Fantozzi et al., 2018
*Calodium hepaticum*	*Calomys venustus*	100	2	2	N/S	N/S	Fantozzi et al., 2018
*Calodium hepaticum*	*Oligoryzomys flavescens*	51	3	5.88	N/S	N/S	Fantozzi et al., 2018
*Calodium hepaticum*	*Oxymycterus rufus*	54	4	7.41	N/S	N/S	Fantozzi et al., 2018
*Capillaria* sp.	*Abrothrix olivacea*	668	107	16.02^**a**^	0.13	0.84	Landaeta-Aqueveque et al., 2007; Riquelme et al., 2021
*Capillaria* sp.	*Abrothrix longipilis*	279	62	22.22	N/A	N/A	Riquelme et al., 2021
*Capillaria* sp.	*Oligoryzomys longicaudatus*	180	12	6.67	N/A	N/A	Riquelme et al., 2021
*Capillaria alainchabaudi*	*Scapteromys aquaticus*	38	5	13.16	N/S	N/S	Robles et al., 2012
*Echinocoleus* sp.	*Scapteromys aquaticus*	130	19	14.62	0.56	3.84	Navone et al., 2009
*Eucoleus* sp.	*Akodon montensis*	104	7	6.73	0.4	6	Panisse et al., 2017
*Gongylonema* sp.	*Abrothrix longipilis*	30	17	56.67	0.33	0.59	Cattan et al., 1992
*Gongylonema* sp.	*Phyllotis darwini*	85	10	11.76	0.99	8.42	Yáñez-Meza et al., 2019
*Guerrerostrongylus zeta*	*Oligoryzomys nigripes*	41	40	97.56^**b**^	32.15	32.95	Digiani et al., 2012; Panisse et al., 2017
*Hassalstrongylus argentinus*	*Holochilus chacarius*	52	36	69.23^**c**^	0.27	0.39	Digiani et al., 2013, Digiani et al., 2015; Gómez-Muñoz et al., 2020
*Hassalstrongylus dollfusi*	*Holochilus chacarius*	37	8	21.62	0.43	2	Serrano et al., 2021
*Hassalstrongylus dollfusi*	*Oligoryzomys flavescens*	45	41	91.11	61.30	67.32	Serrano et al., 2021
*Hassalstrongylus dollfusi*	*Oligoryzomys nigripes*	82	38	46.34	6.16	13.29	Serrano et al., 2021
*Hassalstrongylus mazzai*	*Holochilus chacarius*	31	16	51.61^**d**^	2.68	5.19	Digiani et al., 2015; Gómez-Muñoz et al., 2020
*Heligmosomoides* sp.	*Abrothrix longipilis*	30	17	56.67	0.13	0.24	Cattan et al., 1992
*Heterakis spumosa*	*Abrothrix olivacea*	204	2	0.98^**e**^	0.22	22	Landaeta-Aqueveque et al., 2007; Landaeta-Aqueveque et al., 2018
*Malvinema* sp.	*Scapteromys aquaticus*	130	8	6.15	2.60	42.25	Navone et al., 2009
*Mazzanema fortuita*	*Holochilus chacarius*	52	40	76.92^**f**^	2.73	3.55	Digiani et al., 2013, Digiani et al., 2015; Gómez-Muñoz et al., 2020
*Nematomystes rodentiphilus*	*Scapteromys aquaticus*	130	98	75.38	47.20	62.61	Navone et al., 2009
*Nematomystes rodentiphilus*	*Oxymycterus rufus*	58	1	1.72	0.29	17	Navone et al., 2009
Oxyuroidea sp.	*Phyllotis darwini*	363	4	1.1	N/A	N/A	Carrera-Játiva et al., 2025
Physalopteridae sp.	*Scapteromys aquaticus*	130	26	20	2.3	11.5	Navone et al., 2009
*Physaloptera* sp.	*Abrothrix olivacea*	616	105	17.05^**g**^	0.01	0.04	Riquelme et al., 2021; Carrera-Játiva et al., 2023
*Physaloptera* sp.	*Abrothrix longipilis*	279	62	22.22	N/A	N/A	Riquelme et al., 2021
*Physaloptera* sp.	*Oligoryzomys longicaudatus*	180	12	6.67	N/A	N/A	Riquelme et al., 2021
*Physaloptera* sp.	*Phyllotis darwini*	30	6	20	N/A	N/A	Riquelme et al., 2021
*Physaloptera calnuensis*	*Abrothrix olivacea*	204	27	13.24	0.94	7.07	Landaeta-Aqueveque et al., 2018
*Protospirura* sp.	*Abrothrix longipilis*	30	17	56.67	N/S	N/S	Cattan et al., 1992
*Protospirura* sp.	*Abrothrix olivacea*	54	25	46.3^**h**^	0.56	1.2	Cattan et al., 1992; Carrera-Játiva et al., 2023
*Protospirura numidica criceticola*	*Scapteromys aquaticus*	130	8	6.15	0.2	3.25	Navone et al., 2009
*Protospirura numidica criceticola*	*Akodon azarae*	409	41	10.02^**i**^	0.28	2.76	Sutton, 1989; Miño, 2008; Miño et al., 2012, Miño et al., 2019
*Pseudocapillaria* sp.	*Oxymycterus rufus*	58	8	13.79	0.5	3.63	Navone et al., 2009
*Pterygodermatites* sp.	*Abrothrix olivacea*	225	37	16.44^**j**^	0.42	2.54	Cattan et al., 1992; Landaeta-Aqueveque et al., 2007, Landaeta-Aqueveque et al., 2018
*Pterygodermatites* sp.	*Phyllotis darwini*	134	9	6.72^**k**^	0.15	2.22	Yáñez-Meza et al., 2019; Landaeta-Aqueveque et al., 2018
*Pterygodermatites azarai*	*Akodon azarae*	277	60	21.66^**l**^	0.61	2.8	Miño et al., 2012, Miño et al., 2019
*Pterygodermatites zygodontomys*	*Necromys lasiurus*	40	10	25	0.8	3.2	Gómez-Muñoz et al., 2020
Spirurida sp.	*Akodon azarae*	110	11	10	N/A	N/A	Miño et al., 2020
Spirurina sp.	*Phyllotis darwini*	363	46	12.67	N/A	N/A	Carrera-Játiva et al., 2025
*Stilestrongylus* sp.	*Scapteromys aquaticus*	133	50	37.59^**m**^	20.83	55.42	Digiani et al., 2003; Navone et al., 2009
*Stilestrongylus* sp.	*Akodon azarae*	70	30	42.86	0.04	0.1	Miño et al., 2012
*Stilestrongylus aculeata*	*Akodon montensis*	104	66	63.46	44.8	70.59	Panisse et al., 2017
*Stilestrongylus azarai*	*Akodon azarae*	340	152	44.71^**n**^	N/A	N/A	Durette-Desset and Sutton, 1985; Navone et al., 2009; Miño et al., 2019; Miño et al., 2020
*Stilestrongylus flavescens*	*Oligoryzomys flavescens*	47	27	57.45^**o**^	72.79	126.7	Sutton and Durette-Desset, 1991; Hancke and Suarez, 2018; Navone et al., 2009
*Stilestrongylus stilesi*	*Holochilus chacarius*	69	57	82.61^**p**^	12.57	15.21	Notarnicola et al., 2010; Digiani et al., 2013, Digiani et al., 2015; Gómez-Muñoz et al., 2020
*Stilestrongylus stilesi*	*Necromys lasiurus*	40	37	92.5	120	129.73	Gómez-Muñoz et al., 2020
Strongylida sp.	*Oligoryzomys longicaudatus*	180	12	6.67	N/A	N/A	Riquelme et al., 2021
Strongyloidea sp.	*Phyllotis darwini*	363	23	6.34	N/A	N/A	Carrera-Játiva et al., 2025
*Strongyloides* sp.	*Abrothrix olivacea*	134	2	1.49	N/A	N/A	Carrera-Játiva et al., 2023
*Suttonema* sp.	*Oxymycterus rufus*	58	11	18.97	2.6	13.73	Navone et al., 2009
*Suttonema delta*	*Oxymycterus rufus*	39	12	30.77^**q**^	4	13	Digiani and Durette-Desset, 2003c; Gómez-Muñoz et al., 2020
*Syphacia* sp.	*Abrothrix longipilis*	313	79	25.24^**r**^	0.01	0.03	Cattan et al., 1992; Landaeta-Aqueveque et al., 2018; Riquelme et al., 2021
*Syphacia* sp.	*Abrothrix olivacea*	771	135	17.51^**s**^	1.13	6.46	Cattan et al., 1992; Riquelme et al., 2021; Carrera-Játiva et al., 2023
*Syphacia* sp.	*Oligoryzomys longicaudatus*	191	13	6.81^**t**^	N/A	N/A	Landaeta-Aqueveque et al., 2018; Riquelme et al., 2021; Carrera-Játiva et al., 2023
*Syphacia* sp.	*Phyllotis darwini*	393	10	2.55^**u**^	N/A	N/A	Riquelme et al., 2021; Carrera-Játiva et al., 2025
*Syphacia alata*	*Necromys lasiurus*	40	26	65	17.7	27.23	Gómez-Muñoz et al., 2020
*Syphacia carlitosi*	*Akodon azarae*	344	104	30.23^**v**^	3.13	10.36	Robles and Navone, 2007a; Navone et al., 2009; Miño et al., 2012, Miño et al., 2019; Gómez-Muñoz et al., 2020
*Syphacia carlitosi*	*Akodon montensis*	104	51	49.04	18	36.71	Panisse et al., 2017
*Syphacia hodarae*	*Calomys laucha*	110	40	36.36	1.81	4.98	Rojas-Herrera et al., 2011
*Syphacia kinsellai*	*Oligoryzomys nigripes*	60	22	36.67^**w**^	11.2	30.55	Robles and Navone, 2007b; Panisse et al., 2017; Gómez-Muñoz et al., 2020
*Syphacia obvelata*	*Abrothrix olivacea*	85	6	7.06	2.18	30.83	Landaeta-Aqueveque et al., 2007
*Syphacia phyllotios*	*Phyllotis darwini*	85	11	12.94^**x**^	1.54	11.9	Quentin et al., 1979; Yáñez-Meza et al., 2019
*Tapironema coronatum*	*Akodon montensis*	104	4	3.85	0.1	2.5	Panisse et al., 2017
*Trichofreitasia lenti*	*Akodon montensis*	104	60	57.69	6.2	10.75	Panisse et al., 2017
Trichosomoididae sp.	*Phyllotis darwini*	363	1	0.28	N/A	N/A	Carrera-Játiva et al., 2025
Trichostrongylidae sp.	*Phyllotis darwini*	49	15	30.61	6.35	20.73	Landaeta-Aqueveque et al., 2018
*Trichuris* sp.	*Abrothrix olivacea*	167	18	10.78	0.1	0.94	Carrera-Játiva et al., 2023
*Trichuris* sp.	*Phyllotis darwini*	363	10	2.75	N/A	N/A	Carrera-Játiva et al., 2025
*Trichuris laevitestis*	*Akodon azarae*	426	112	26.29	N/A	N/A	Miño et al., 2020
*Trichuris laevitestis*	*Scapteromys aquaticus*	315	11	3.49^**y**^	0.28	8.09	Suriano and Navone, 1994; Robles and Navone, 2006; Navone et al., 2009
*Trichuris navonae*	*Akodon montensis*	133	103	77.27^**z**^	3.70	4.78	Robles, 2011; Robles et al., 2014b; Panisse et al., 2017
*Trichuris pardinasi*	*Phyllotis darwini*	49	2	4.08	0.8	19.5	Landaeta-Aqueveque et al., 2018

P (%), prevalence; _M_A, mean abundance; _M_I, mean intensity; N/S, not specified; N/A, not applicable. Bold superscript numbers next to ‘P (%)’ values indicate the corresponding 95% confidence interval (95% CI), detailed as follows.

a (13.22–18.82); ^b^(92.9–102.22); ^c^(56.18–82.29); ^d^(33.73–69.49); ^e^(0–2.33); ^f^(65–88.84); ^g^(13.98–20.12); ^h^(32.72–59.89); ^i^(7.04–12.99); ^j^(11.44–21.44); ^k^(2.42–11.01); ^l^(16.77–26.56); ^m^(29.34–45.84); ^n^(39.39–50.04); ^o^(43.43–71.46); ^p^(73.06–92.17); ^q^(16.25–45.28); ^r^(20.35–30.13); ^s^(14.68–20.34); ^t^(3.22–10.40); ^u^(0.99–4.1); ^v^(25.45–35.02); ^w^(24.50–48.83); ^x^(5.62–20.26); ^y^(1.45–5.53); ^z^ (70.13–84.41).

## Discussion

Here, we summarize the current knowledge on the diversity of nematodes inhabiting the gastrointestinal tract and accessory organs of cricetid rodents in Argentina and in Chile. Our analysis of 53 studies spanning five decades reveals 92 nematode *taxa* parasitizing 33 cricetid species across 4400 individuals examined, representing the first comprehensive regional assessment of these host–parasite associations in southern SA. This review provides an overview of infection indicators and a geographic perspective that enables identification of regional-scale patterns, while synthesizing the methodological approaches employed in the reviewed studies. Moreover, the review identifies important research gaps. For instance, only three of the 53 reviewed studies (5.66%) used molecular tools to complement morphological identification of nematodes (Robles et al., 2014b, 2018; Fantozzi et al., 2018), underscoring the need to expand integrative taxonomic approaches that combine morphological and molecular evidence.

### Rodent sampling and representation

Rodent representation in the reviewed literature is markedly unequal, showing taxonomic and geographic sampling bias that should be carefully considered when interpreting patterns of nematode diversity. The cricetid species with the highest numbers of examined individuals (i.e., *A. olivacea, A. azarae, P. darwini, S. aquaticus,* and *A. longipilis*) are characterized by high ecological plasticity, broad habitat uses, and omnivorous diets (Patton et al., 2015; Miño et al., 2020). These traits likely make them more abundant, more detectable and easier to trap across range of environments, which, in turn, increases their representation in parasitological surveys.

Geographic bias further reinforces this uneven host representation. Argentina accounted for most studies (41 articles; 77.36%), including extensive samplings of *A. azarae* (*n* = 775), whereas Chile, despite fewer publications (12 articles; 22.6%), reported an elevated number of examined *A. olivacea* individuals (*n* = 1000). This pattern likely reflects not only differences in capture success, detectability, and local abundance, with *A. olivacea* being more frequently captured than other cricetid species in Chile (Gaona et al., 2024), but also the specific objectives and logistical constraints of the studies. In several cases, sampling was deliberately focused on filling geographical and host-related knowledge gaps, targeting areas with scarce previous records and rodent species of ecological or epidemiological interest.

Importantly, this overrepresentation does not align proportionally with the species’ geographic distributions: *A. olivacea* and *A. longipilis* are restricted to Argentina and Chile, and *P. darwini* to specific regions of central and northern Chile, whereas *A. azarae* and *S. aquaticus* range broadly across SA (e.g., Patton et al., 2015; Miño et al., 2020; Teta et al., 2025). The frequency with which these hosts appear in the reviewed literature, therefore, seems to reflect sampling intensity and capture success more than their actual biogeographic ranges. This disparity complicates direct comparisons of nematode diversity among countries and host species, as observed differences may partly stem from uneven sampling effort instead of genuine ecological or biogeographic processes.

The observed variation in nematode diversity across regions is probably influenced by ecological dynamics and sampling imbalances. Environmental factors, such as land use changes, weather elements (e.g., humidity and temperature) and vegetation structure, are known to influence rodent and nematode communities (Chaisiri et al., 2017). Nevertheless, the lack of standardized information on whether study sites are human-modified or relatively pristine environments limits our ability to formally assess how these variables affect host–parasite associations. Without this classification, differences in nematode diversity may be confounded by unmeasured habitat effects, masking the underlying ecological processes.

These limitations underscore the importance of standardized reporting of environmental and sampling data in future studies. Such information would enable comparisons across disturbance intervention gradients and enhance our comprehension of how ecological context may modulate nematode transmission, survival, and species richness in cricetid rodents within Argentinian and Chilean ecosystems (Miño et al., 2019; Grandón-Ojeda et al., 2022).

### Diversity patterns of nematode–cricetid rodent associations

A total of 92 nematode *taxa* have been recorded in cricetid rodents, of which 64 were identified to the species level. Among them, *C. hepaticum* stands out due to its occurrence in six different rodent species. This nematode is widely distributed across diverse geographic regions and is commonly reported in rodents of the family Muridae (Torres and Gonzalez, 1972; Hancke et al., 2011; Duque et al., 2012; Walker et al., 2016; Liu et al., 2025), although it has also been occasionally documented in Cricetidae (Fantozzi et al., 2018, 2022; Liu et al., 2025). Its broad host range, which includes humans, points to its public health relevance (Fuehrer et al., 2011; Fuehrer, 2014a, 2014b; Fantozzi et al., 2018, 2022). However, the relatively sporadic records in cricetids suggest that further research is needed to clarify its transmission dynamics and to identify the factors underlying its occurrence across different rodent groups and environments (Cole and Viney, 2018).

Other nematodes, however, showed recurrent associations with specific cricetid hosts. In particular, we identified *St. azarai, Sy. carlitosi,* and *T. laevitestis* as the most frequent nematodes in cricetid rodents. Notably, these parasitic worms were regularly found in *A. azarae*, one of the most extensively studied rodent hosts (Durette-Desset and Sutton, 1985; Suriano and Navone, 1994; Robles and Navone, 2006, 2007a; Navone et al., 2009; Miño et al., 2012, 2019; Gómez-Muñoz et al., 2020). The high occurrence of *Sy. carlitosi* in *A. azarae* may indicate a relatively well-tolerated parasitic relationship (Robles, 2010). Nevertheless, in the absence of studies evaluating their effects at the individual level (such as on host condition, survival and reproduction) and at the population level, including effects on abundance and dynamics, it remains difficult to determine the severity of infection or its overall impacts for host populations (Kristan, 2004; Maroli, 2014; Fantozzi et al., 2019; Palavecino et al., 2023).

Variation in nematode infection levels among rodent species is likely influenced by multiple interacting factors, such as behaviour, immune and physiological susceptibility, nematode life-history strategies and environmental conditions (Kristan, 2004; Pakdeenarong et al., 2014; Fantozzi et al., 2019). In addition, methodological aspects may substantially influence the detection and estimation of infection levels. Sampling design (e.g., effort per site, number of nights, spatial coverage), type of traps and baits used, study objectives and the years and seasons sampled can all affect the possibility of captured infected versus uninfected individual (Monge, 2010; Farid et al., 2021; Carrera-Játiva et al., 2023, 2025).

Accordingly, the nematode diversity patterns associated with cricetid rodents synthetized here should be viewed as preliminary patterns, given the inherent biases arising from the wide variability of both biological and methodological factors involved. These findings provide a starting point for future research to delve deeper into host–parasite dynamics and more comprehensively characterize rodent parasite fauna (Poulin, 2007). Rodent species that harbour a greater diversity of nematodes may occupy broader habitats or have more diverse diets, thereby increasing their exposure to a wider array of parasites (Pardiñas et al., 2006; Robles, 2010).

In the analysed dataset, for instance, *A. olivacea* and *A. azarae* showed the highest nematode richness (e.g., Landaeta-Aqueveque et al., 2007, 2018; Miño et al., 2012, 2019, 2020; Yáñez-Meza et al., 2019; Gómez-Muñoz et al., 2020; Riquelme et al., 2021; Serrano et al., 2021; Carrera-Játiva et al., 2023), which may be explained by their wide geographic distribution and ecological flexibility. In contrast, rodent species with more restricted distributions, such as *A. sanborni, C. callidus, C. laucha, C. venustus, D. dorsalis, L. micropus, N. squamipes,* and *O. quaestor*, tended to show lower nematode richness (Denke and Murua, 1977; Durette-Desset and Murua, 1979; Sutton et al., 1980; Rojas-Herrera et al., 2011; Digiani and Kinsella, 2014; Panisse et al., 2017; Fantozzi et al., 2018). This pattern may reflect their more limited habitat range and/or lower population densities, potentially reducing exposure to distinct parasite species. Furthermore, small sample sizes for some host species probably reduce detection and may underestimate true nematode richness.

The apparent specificity of certain nematodes to a single rodent species in particular studies could reflect reciprocal evolutionary adaptations or ecological constraints. However, it may also result from limited sampling effort that fails to detect these parasites in alternative hosts (Hoberg, 1992; Hoberg et al., 1997; Nieberding and Morand, 2006; Robles, 2010). In some cases, such patterns of specificity may be associated with restricted host distributions, low host density or logistical challenges in sampling. Therefore, new host records and apparent host absence contribute to the understanding of host–parasite relationship specificity but should always be interpreted cautiously and within the context of sampling coverage and host availability (Poulin, 2007).

Beyond contemporary surveys, paleoparasitological studies based on rodent burrows and coprolites from Argentina and Chile have documented nematodes associated with small mammal communities across historical and prehistoric timescales (Tietze et al., 2020; Oyarzún-Ruiz et al., 2021). These findings suggest that several host–parasite associations observed today may have deep temporal continuity, providing important context for present-day patterns of occurrence and host range. Although paleoparasitological data were not included in our synthesis, their integration into future comparative analyses could offer a broader temporal framework to evaluated the stability, turnover, and biogeographic history of nematode–cricetid assemblages in the Southern Cone (Fugassa, 2014; Littlewood and de Baets, 2015; Oyarzún-Ruiz et al., 2021).

### Infection metrics: interpreting P, _M_A and _M_I

The infestation metrics recorded in this review reveal distinct ecological patterns of nematode–cricetid rodent associations. The very high P of *G. zeta* in *O. nigripes* (97.56%), *St. stilesi* in *N. lasiurus* (92.5%), and *H. dollfusi* in *O. flavescens* (91.11%) indicates that infection is nearly ubiquitous within these host populations. Such consistently high P values are characteristic of well-established host–parasite associations marked probably by efficient transmission and frequent exposure within host microenvironments (Marcogliese, 2004; Poulin, 2007, 2010; Kersul et al., 2020; Scott, 2023). These host species are, therefore, likely to function as core host reservoirs that maintain local transmission of these nematodes (Haydon et al., 2002; Hallmaier-Wacker et al., 2017).

On the other hand, the highest values of _M_A and _M_I, recorded for *St. flavescens* in *O. flavescens* (_M_A = 72.79 and _M_I = 126.7) and *St. stilesi* in *N. lasiurus* (_M_A = 120 and _M_I = 129.73), suggest that, in these nematode-rodent associations, infected rodents harbour extremely heavy worm burdens (_M_A and _M_I > 70–120). Ecologically, the combination of high P and high _M_I implies that many individuals contribute substantially to environmental contamination with infective stages, potentially reinforcing transmission cycles and promoting strongly aggregated parasite distributions typical of macroparasites (Marcogliese, 2004; Poulin, 2007, 2010; Scott, 2023). Conversely, nematodes with high P but comparatively lower _M_I are likely to produce more widespread yet lighter infections, potentially leading to different consequences for host condition and for the stability and persistence of nematode-rodent associations (Poulin, 2007, 2010; Scott, 2023).

Considering P, _M_A, and _M_I jointly, therefore, provides insight into how infestation is structured within host populations, as opposed to just prevalence. High P combined with high _M_I points to associations in which infection is both common and intense, whereas high P with moderate _M_I, or lower P with high _M_I, suggests more heterogeneous and potentially highly aggregated patterns of parasite distribution among hosts (Bush et al., 1997; Poulin, 2007, 2010; Scott, 2023). These patterns are likely shaped by host ecology traits (e.g., habitat use, social behaviour, diet), host immune responses, parasite life cycles, and environmental conditions (Kristan, 2004; Poulin, 2010; Pakdeenarong et al., 2014; Scott, 2023). Taken together, these results underscore that prevalence alone is insufficient to fully characterize the ecology of and transmission dynamics of nematode infections within these rodent communities.

### Methodological considerations and integrative taxonomy

Most studies relied on necropsy (e.g., Babero et al., 1976; Durette-Desset et al., 1976; Denke and Murua, 1977; Durette-Desset and Murua, 1979; Quentin et al., 1979; Sutton, 1979, 1989; Sutton et al., 1980; Rojas and Digiani, 2003; Robles and Navone, 2006, 2007a, 2007b, 2010; Robles et al., 2006, 2012, 2014a, 2014b, 2018; Digiani and Durette-Desset, 2007; Robles, 2011; Rojas-Herrera et al., 2011; Digiani and Kinsella, 2014; Castillo et al., 2016; Panisse and Digiani, 2018; Chemisquy et al., 2021; Serrano et al., 2021), which is a direct and generally effective method for detecting and identifying nematodes. However, reliance solely on necropsy may introduce detection bias, particularly against very small nematode species, low-intensity infections or parasites located in anatomical sites that are not routinely or exhaustively examined.

To overcome these limitations, necropsy-based approaches could be complemented with coprological concentration techniques and faecal molecular diagnostics like PCR (Fantozzi et al., 2018; Miño et al., 2020; Riquelme et al., 2021; Carrera-Játiva et al., 2023, 2025). The combined use of these methods may increase detection sensitivity, particularly for small species, larval stages and infections in less accessible tissues, thereby reducing the risk of underestimation. Remarkably, in most reviewed studies, nematode searches were restricted to the gastrointestinal tract, with accessory organs only occasionally examined (Supplementary Table S1).

In fact, among the reviewed papers, only one study (1.89%) examined rodent livers for nematode infection (Fantozzi et al., 2018), reporting the presence of *C. hepaticum*. In contrast, no study evaluated the pancreas; consequently, no records of nematodes in this organ were identified. Although no pancreatic infections were identified in rodents in our review, isolated evidence from other mammals (Bain and Junker, 2013; Barbosa-Valenzuela et al., 2021; Regmi et al., 2024; Chang et al., 2025) and the anatomical and physiological continuity between the pancreas and the gastrointestinal tract (Shaik et al., 2025) suggest that nematodes may occasionally reach and establish in this organ under particular circumstances, such as high parasite loads, aberrant migration or systemic dissemination (Bain and Junker, 2013; Barbosa-Valenzuela et al., 2021; Regmi et al., 2024; Chang et al., 2025). Therefore, the low number of liver records and the lack of pancreatic records in cricetid rodents probably reflect limited examination rather than true absence. The routine inclusion of accessory organs in examination protocols could improve the detection of *taxa* that might otherwise remain overlocked (Roeber et al., 2013).

Nematode identification has traditionally relied on morphological traits, such as body length, reproductive structures and cephalic and caudal features (e.g., Sutton, 1989; Robles et al., 2006; Digiani and Kinsella, 2014; Serrano et al., 2021). While morphology remains a key, it may be insufficient when closely related *taxa* exhibit limited diagnostic differentiation (Oliveira et al., 2011; Bradbury et al., 2022). In such cases, integrative approaches that combine molecular tools with morphological analysis may provide a more robust framework for nematode identification, particularly for resolving cryptic diversity (Roeber et al., 2013; Cháves-González et al., 2022).

Among the reviewed studies, only three (Robles et al., 2014b, 2018; Fantozzi et al., 2018) applied an integrative approach by analysing adult nematodes using both morphological and molecular techniques, underscoring the still limited adoption of integrative taxonomy in parasitic helminth systematics. Expanding the application of integrative taxonomy will be key for resolving taxonomic ambiguities and improving our understanding of host specificity, coevolution and zoonotic potential of these parasites.

### Conclusions and implications

This systematic review documents a high diversity of nematodes infecting cricetid rodents in Argentina and Chile, while simultaneously revealing important knowledge gaps related to taxonomic resolution, geographic coverage, and host representation. The nematodes *C. hepaticum, St. azarai, Sy. carlitosi,* and *T. laevitestis* were identified; among them, *C. hepaticum* showed the broadest host range and the greatest potential epidemiological relevance.

The marked spatial heterogeneity in nematode richness and composition, together with nematode–cricetid rodent associations characterized by very high P and parasite burdens, points to complex ecological dynamics that may have potential consequences for host health, nematode transmission, and host population regulation. However, it is important to interpret infection metrics within their ecological and methodological contexts, rather than relying solely on prevalence estimates.

Methodologically, the predominance of necropsy-based studies and morphological-centred identification, coupled with the limited use of molecular tools, constrains the detection of cryptic diversity and a more refined understanding of host specificity, parasite distribution and evolutionary relationships. Expanding the application of integrative taxonomy approaches will be essential to advance helminth systematics and ecological inference.

Finally, in light of the rapidly growing research on wildlife parasitology in SA, it is important to note that this review synthesizes evidence available up to 10 January 2026. Studies published thereafter will likely broaden the known host–parasite spectrum, refine taxonomic frameworks, and provide deeper ecological and epidemiological insights. As such, the present synthesis should be taken as a dynamic baseline that can guide future research aimed at improving our understanding of nematode–cricetid associations in the Southern Cone of SA.

## Supporting information

10.1017/S0031182026102029.sm001 Ardila et al. supplementary material Ardila et al. supplementary material

## Data Availability

The data supporting the findings of this study are contained within the manuscript or supplementary material. The raw data are available by the corresponding author when requested.

## Supplementary material

The supplementary material for this article can be found at https://doi.org/10.1017/S0031182026102029.

## Figure 1 Long description

The flowchart illustrates the PRISMA method for study selection in a systematic review. It begins with the identification phase, showing a total of 3869 records identified from databases: 1285 from PubMed, 584 from WoS, 260 from Scopus, 18 from SciELO and 1722 from Google Scholar. Seventy-two duplicate records are removed, leaving 3797 unique records. In the screening phase, titles and abstracts are reviewed, excluding 3675 records, resulting in 122 studies eligible for full-text review. During the eligibility phase, 79 studies are excluded, leaving 43 studies that meet the inclusion criteria. An additional 10 studies are found through indirect search, leading to a total of 53 studies included in the final dataset.

Navigate back to Figure 1.

## Figure 2 Long description

The map of Argentina and Chile illustrates the geographical distribution and local composition of cricetid rodent genus richness. Surveyed localities are marked with pie charts representing the relative proportion of rodent genera recorded at each site. The pie charts vary in size, proportional to the total number of rodent genera at each locality, with a reference scale indicating sizes for 5, 10 and 15 genera. The map includes regions such as Coquimbo, Valparaiso, Metropolitana, Maule, La Araucania, Los Rios, Los Lagos in Chile and provinces like Salta, Formosa, Misiones, Corrientes, Entre Rios, Buenos Aires, La Pampa, Mendoza, San Luis, San Juan, La Rioja, Catamarca, Chaco, Santa Fe, Cordoba in Argentina. The legend identifies rodent genera with color-coded labels: Abrothrix, Akodon, Brucepattersonius, Calomys, Deltamys, Euryoryzomys, Geoxus, Graomys, Holochilus, Irenomys, Loxodontomys, Necromys, Neotomys, Oligoryzomys, Oxymycterus, Phyllotis, Reithrodon, Scolomys, Scapteromys, Sooretamys, Thaptomys. The inset map shows the study area within South America, highlighting Argentina and Chile. The pie charts indicate the highest rodent genus richness in Buenos Aires and Misiones provinces in Argentina and Los Lagos region in Chile.

Navigate back to Figure 2.

## Figure 3 Long description

The map of Argentina highlights surveyed localities, showing nematode richness in cricetid rodents. Each province is marked with pie charts representing the relative proportion of nematode species detected. The size of each pie chart corresponds to the total number of nematode taxa found at that site. Buenos Aires and Misiones provinces have the highest richness, with 28 and 23 taxa respectively. Other provinces like La Pampa, San Juan, Cordoba and Santa Fe show lower richness with one taxon each. The legend lists nematode taxa with corresponding colors used in the pie charts. The map includes a scale bar indicating distances in kilometers and a reference for pie chart sizes, showing numbers 5, 10 and 15. The inset map shows the location of Argentina within South America, with a focus on the surveyed regions.

Navigate back to Figure 3.

## Figure 4 Long description

A thematic map of Chile with north at top. The main map shows the long north to south outline of Chile with internal region boundaries and region labels: Coquimbo, Valparaiso, Metropolitana, Maule, La Araucania, Los Rios and Los Lagos. Surveyed localities are marked by filled circular symbols. Several of these locality symbols have an adjacent pie chart. The pie slices correspond to nematode taxa listed in the legend titled, Nematode taxa in Cricetid rodents. A separate size reference titled, Number of nematode taxa, shows three example circle sizes labeled 5, 10 and 15 and a larger example labeled 19. A scale bar is shown with labels 0 and 100 km. An inset map at upper right shows South America with a boxed area and a highlighted strip along the west side corresponding to Chile. A second inset below it shows a larger view of the same highlighted strip. Pie charts appear next to multiple localities across the country, including in the northern labeled regions and in the central and southern labeled regions. The largest pie chart is located in the northern part of the main map near the Coquimbo label. Smaller pie charts are shown near the Valparaiso and Metropolitana labels and additional pie charts are shown farther south near the Los Rios and Los Lagos labels.

Navigate back to Figure 4.

## Figure 5 Long description

A) Map of Argentina and Chile showing nematode taxon richness by province or region. Richness ranges from 1 to 3, 4 to 6, 7 to 9 and 10 to 13. Surveyed localities are marked with red numbers: 1. Misiones, 2. Buenos Aires, 3. Corrientes, 4. Coquimbo, 5. Metropolitana, 6. Formosa, 7. Catamarca, 8. Valparaiso, 9. Los Lagos, 10. La Rioja, 11. Los Rios, 12. San Luis, 13. Entre Rios, 14. Cordoba, 15. Salta, 16. La Araucania, 17. Maule. B) Bar chart showing frequency of nematode occurrence in rodents, with bars colored by rodent species: Akodon azarae, Akodon montensis, Oligoryzomys flavescens, Scapteromys aquaticus and others. Nematode taxa include Syphacia, Stilestrongylus and Trichuris. C) Bar charts showing the number of rodent host species associated with each nematode genus: Stilestrongylus, Syphacia and Trichuris. Each chart lists nematode species and their host range, with Stilestrongylus azarai, Syphacia carlitosi and Trichuris laevitestis being prominent species.

Navigate back to Figure 5.

## Table 1 Long description

Summary of parasitological studies is referenced, comparing sample types and diagnostic techniques used to detect gastrointestinal and accessory-organ nematodes in cricetid rodents from Argentina and Chile. No rows, columns, counts, or study entries were provided, so specific techniques, sample categories, and any differences between countries cannot be identified. Without the table body, it is not possible to report which methods were most common, which sample types were most frequently examined, or whether certain techniques were linked to particular organs or taxa. Provide the table data (headers and all cells) to generate accurate, WCAG-compliant that includes key comparisons and trends.

Navigate back to Table 1.

## Table 2 Long description

Nematode infection metrics in cricetid rodents from Argentina and Chile are intended to be summarized by parasite taxon and host organ, using prevalence, mean abundance, and mean intensity. The prompt references confidence intervals tied to prevalence values, but the underlying table entries are missing. Because no rows or numbers are available, it is not possible to report which nematodes were recorded, which hosts or organs were most affected, or how infection levels compare across species or locations. Any interpretation about trends, highest prevalence, or differences between countries would be speculative without the actual data. Provide the table body to generate accurate describing the main results and comparisons.

Navigate back to Table 2.
